# *KCNMA1* Expression Is Downregulated in Colorectal Cancer via Epigenetic Mechanisms

**DOI:** 10.3390/cancers11020245

**Published:** 2019-02-19

**Authors:** Maria Sofia Basile, Paolo Fagone, Katia Mangano, Santa Mammana, Gaetano Magro, Lucia Salvatorelli, Giovanni Li Destri, Gaetano La Greca, Ferdinando Nicoletti, Stefano Puleo, Antonio Pesce

**Affiliations:** 1 Department of Biomedical and Biotechnological Sciences, University of Catania, Via S. Sofia 89, 95123 Catania, Italy; sofiabasile@hotmail.it (M.S.B.); kmangano@unict.it (K.M.); ferdinic@unict.it (F.N.); 2 IRCCS Centro Neurolesi “Bonino-Pulejo”, Strada Statale 113, C.da Casazza, 98124 Messina, Italy; santa84ma@libero.it; 3 Department of Medical and Surgical Sciences and Advanced Technology “G.F. Ingrassia”, University of Catania, Via Santa Sofia 86, 95123 Catania, Italy; g.magro@unict.it (G.M.); lucia.salvatorelli@unict.it (L.S.); g.lidestri@unict.it (G.L.D.); glagreca@unict.it (G.L.G.); spuleo@unict.it (S.P.); nino.fish@hotmail.it (A.P.)

**Keywords:** KCNMA1, Colorectal Cancer, epigenetics, DNA methylation, mir-17-5p, mir-31, mir-211

## Abstract

*KCNMA1* is a gene located at 10q22 that encodes the pore-forming α-subunit of the large-conductance Ca^2+^-activated K^+^ channel. *KCNMA1* is down-regulated in gastric carcinoma tumors, through hypermethylation of its promoter. In the present study, we have evaluated the expression levels of *KCNMA1* both in a mouse model of Colorectal Cancer (CRC) and in human CRC samples. Additionally, epigenetic mechanisms of KCNMA1 gene regulation were investigated. We observed a significant down-regulation of *KCNMA1* both in a human and mouse model of CRC. No differences in *KCNMA1* levels were, however, observed at different TNM stages. We also wanted to determine whether the modulation in KCNMA1 was dependent on epigenetic mechanisms. A statistically significant inverse correlation between KCNMA1 expression and mir-17-5p levels was observed in patients with CRC. Furthermore, in the tumor samples, we found a significant hypermethylation of the promoter, in the loci cg24113782 and cg25655799, compared to healthy tissue. Overall, our data suggest the possible use of KCNMA1 as a therapeutic target in the early stages of CRC.

## 1. Introduction

*KCNMA1* is a gene encoding the Calcium-Activated Potassium Channel Subunit Alpha-1, also known as the Large Conductance Calcium-Activated Potassium Channel, Subfamily M, Alpha Member 1 (KCa1.1), which represents high voltage-activated channel conductance for potassium ions [1]. *KCNMA1* is located at chromosome 10 (10q22.3) and chromosome 14 in the human and murine genome, respectively. The channel consists of four subunits that self-assemble to form homo-tetramers and is located in the endoplasmic reticulum, in the Golgi apparatus, and in the cellular plasma membrane [1]. The channel is involved in different tumor processes, from cell proliferation to apoptosis, and response to hypoxia and to chemotherapeutic agents. Growing evidence indicates that K^+^ channels may be involved in the oncogenesis process. Amplification of the *KCNMA1* gene was observed in 16% of advanced prostate tumors [2] and an up-regulation of its expression was observed in breast carcinoma [3,4,5], prostate cancer [6], glioblastoma [7], and cervical cancers [8]. In contrast, *KCNMA1* is down-regulated or silenced in primary cells and in gastric carcinoma cell lines (MGC-803, BGC-823, MKN-82, SGC-7901), through hyper-methylation of its promoter, in particular, the CpG island, cg24113782 [9]. A role for miRNAs, in particular mir-17-5p, mir-31, and mir-211, in the regulation of KCNMA1 expression has also been determined in different tumors, including ovarian cancer [10], pleural mesothelioma [11], and cutaneous melanoma [12].

The primary aim of this study was to define the modulation of the *KCNMA1* gene, both in a mouse model of colorectal cancer (CRC) and in human CRC samples. To this aim, we have used the well-established model of CRC, induced by the administration of dextran sodium sulfate (DSS)/azoxymethane (AOM), which rapidly recapitulates the aberrant crypt foci-adenoma-carcinoma sequence that occurs in human CRC [13,14]. The secondary aim of the study was to determine whether the modulation of this gene may correlate with the disease stage or the Overall Survival (OS) of the patients. Finally, we have evaluated the potential epigenetic mechanisms involved in the regulation of KCNMA1 expression in CRC.

## 2. Results

### 2.1. KCNMA1 Expression Is Modulated in Preclinical Models of Ulcerative Colitis (UC) and UC-Associated CRC

**Figure 1:**
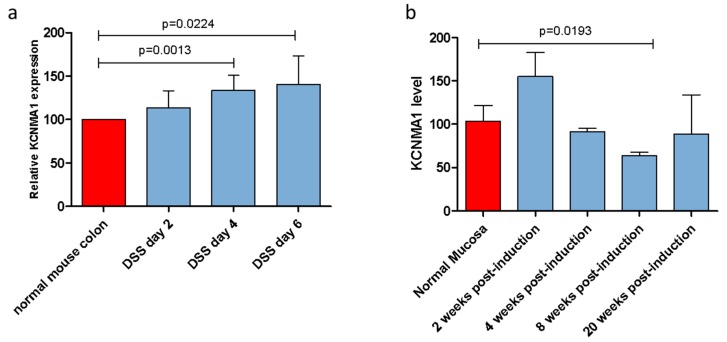
KCNMA1 is modulated in murine models of ulcerative colitis (UC) and colorectal cancer. (**a**) Transcriptional levels of KCNMA1 in a murine model of UC induced by the administration of 3% dextran sodium sulfate (DSS) in drinking water were determined by Real-Time PCR; (**b**) expression of KCNMA1 in the DSS/azoxymethane model was determined by Real-Time PCR at different time points (0, 2, 4, 8, and 20 weeks from disease induction) (*n* = 5–6 animals per group). The expression level is presented as percentage of increase as compared to control (normal mucosa) group, which was arbitrarily set to 100. ANOVA followed by Bonferroni post hoc test was used to assess statistical differences among groups.

In the UC model induced by DSS administration, a significant increase in *KCNMA1* levels was observed in the inflamed colonic mucosa, starting from day 4 (*p* = 0.0013) up to day 6 post-induction (*p* = 0.0224) (Figure 1a). Similarly, a 49.4% increase in *KCNMA1* levels was observed in the second week after the DSS/AOM administration in the colorectal model (Figure 1b). In contrast, an important reduction of *KCNMA1* expression levels in the colorectal mucosa can be observed starting from the fourth week of DSS/AOM administration, reaching statistical significance in the mucosa with high degree dysplasia (eighth week) (*p* = 0.01923). 

At the 20th week post-induction, there was a trend toward lower levels of *KCNMA1* levels in samples from diseased animals as compared to those observed in the healthy mucosa of the controls (Figure 1b).

### 2.2. KCNMA1 Levels Are Reduced in Human CRC

**Figure 2:**
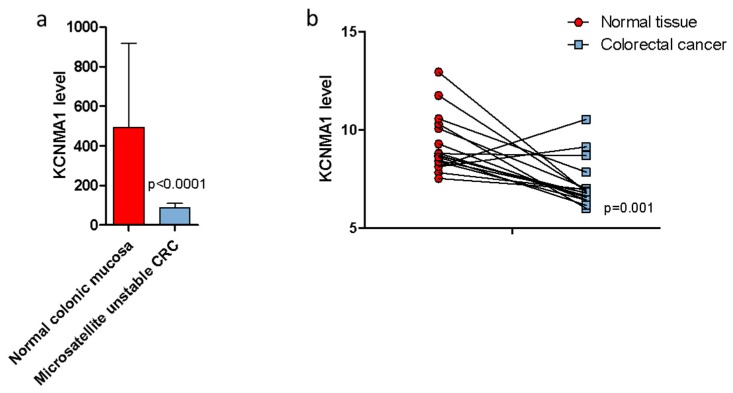
KCNMA1 expression is modulated in colorectal cancer (CRC). (**a**) KCNMA1 levels were determined in colonic mucosa of healthy subjects (*n* = 15) and in patients with microsatellite unstable CRC (*n* = 34); (**b**) and on neoplastic and normal tissue of each patient (*n* = 17). Unpaired (**a**) and paired (**b**) two-tailed Student’s T test was applied to assess statistical differences between groups.

In order to confirm the results obtained in the DSS/AOM murine model, the microarray datasets GSE24514 and GSE32323 were analyzed. As shown in Figure 2, significantly lower levels of *KCNMA1* (*p* < 0.001) were observed in patients with colorectal adenocarcinoma bearing microsatellite instability (MSI) as compared to healthy patients (Figure 2a), and in neoplastic tissue as compared to healthy mucosa (Figure 2b).

**Figure 3:**
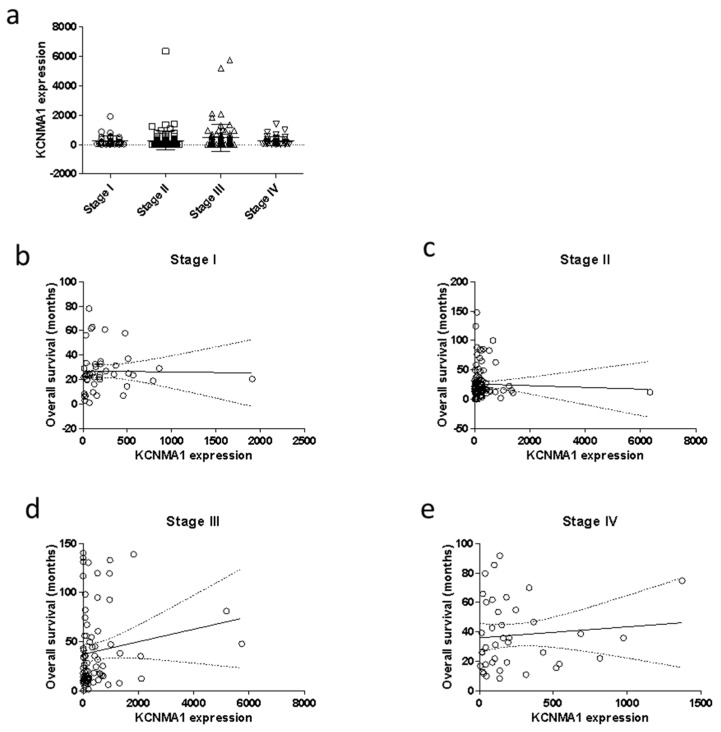
Modulation of the transcriptional levels of KCNMA1 in relation to TNM stage and overall survival (OS). Data regarding the levels of KCNMA1 in patients (*n* = 282) with CRC were obtained from the TGCA database, through the cBioPortal for Cancer Genomics (http://www.cbioportal.org/) website. Statistical differences among groups were evaluated by applying ANOVA test. The correlation between KCNMA1 levels and OS was calculated by using the non-parametric Spearman’s test. (**a**) KCNMA1 expression levels in CRC samples at different disease stages; (**b**) correlation between KCNMA1 expression and OS in samples of CRC at stage I; (**c**) correlation between KCNMA1 expression and OS in samples of CRC at stage II; (**d**) correlation between KCNMA1 expression and OS in samples of CRC at stage III; (**e**) correlation between KCNMA1 expression and OS in samples of CRC at stage IV;

No correlation was found between the transcriptional levels of *KCNMA1* and the tumor stage (Figure 3a). We have also performed a correlation analysis between KCNMA1 and the OS, stratifying the patients’ population for stage of the disease. As shown in Figure 3, there is no significant correlation between KCNMA1 and survival, although a trend of correlation (*p* = 0.1839) can be observed in Stage III patients. (Figure 3d).

Analysis of the genetic mutations in the DNA-sequenced cohort of CRC patients from the TGCA database revealed that only 3.54% of patients with CRC bears a mutation in the *KCNMA1* gene (http://www.cbioportal.org/)), including one case of in-frame deletion and nine cases of missense mutations, as shown in Table 1. These results therefore suggest that the altered levels of KCNMA1 gene expression in patients with CRC are not determined by the systematic presence of genetic mutations.

**Figure 4:**
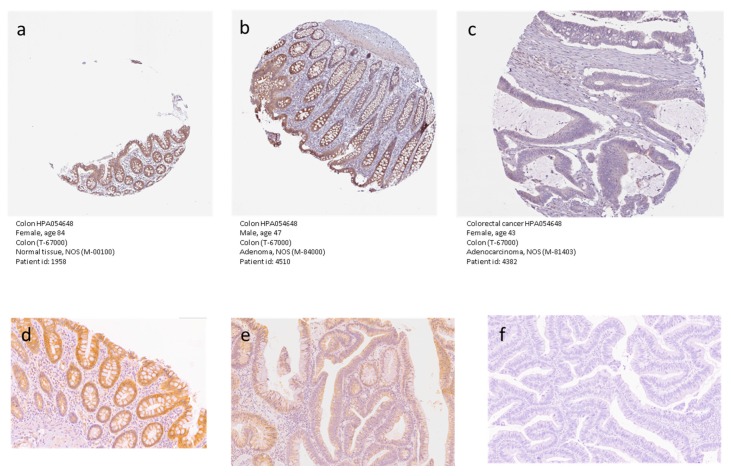
Immunohistochemical analysis for KCNMA1 protein expression in samples of normal colonic mucosa (**a**), adenoma (**b**), and adenocarcinoma (**c**). The images obtained from The Human Protein Atlas database (https://www.proteinatlas.org/pathology). Immunohistochemical analysis for KCNMA1 protein expression in samples of normal colonic mucosa (**d**), tubule-villous adenoma with low-grade dysplasia (**e**), and adenocarcinoma (**f**). Magnification for all images: 20×.

In order to validate the data from the preclinical models and the bioinformatic analysis, we first consulted the freely accessible database, The Human Protein Atlas (https://www.proteinatlas.org/). According to the database, endothelial cells of the normal colon samples show a low staining intensity for *KCNMA1*, while the glandular and the peripheral nerve/ganglion cells have a medium staining positivity for the HPA054648 antibody staining. As compared to normal colon samples, lower levels of KCNMA1 expression were observed in CRC samples, which showed a high staining intensity in zero out of 12 samples, medium intensity in one out of 12 samples, low intensity in six out of 12 samples, and no positivity in five out of 12 samples (https://www.proteinatlas.org/). Representative pictures of normal and CRC samples are presented in Figure 4. We have also validated the data obtained from The Human Protein Atlas by performing immunohistochemical analysis in three samples comprising normal colonic mucosa (a), tubule-villous adenoma with low-grade dysplasia (b), and adenocarcinoma (c). Protein expression was present in normal epithelium, reduced in dysplastic epithelium, and completely lost in neoplastic epithelium.

### 2.3. MiRNA Analysis and Methylation Profile of KCNMA1

**Figure 5:**
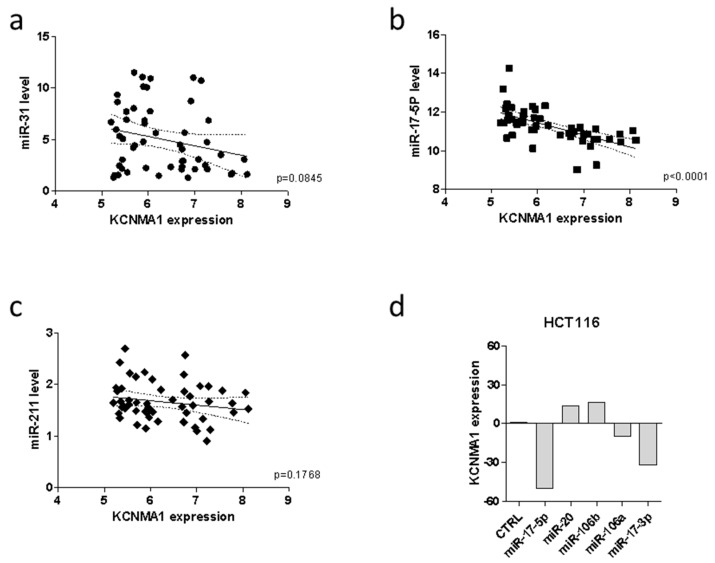
MiRNAs regulating KCNMA1 expression. Correlation between KCNMA1 expression levels and miR-31 (**a**), miR-17-5p (**b**), and miR-211 (**c**) in CRC patients (*n* = 31), as determined in the GSE35834 dataset; (**d**) modulation of KCNMA1 expression upon transfection of HCT116 cells with miRNAs belonging to the miR-106b family, as determined in the GSE14831 dataset.

Previous reports have shown that epigenetic mechanisms are directly involved in the regulation of KCNMA1 expression in a variety of tumors. To evaluate whether KCNMA1 modulation in CRC could be associated with specific miRNA profiles, the GSE35834 microarray dataset was interrogated. A statistically significant inverse correlation was observed between KCNMA1 expression and mir-17-5p (*p* < 0.0001), while only a trend of inverse correlation was observed for mir-31 (*p* = 0.0845) in patients with CRC (Figure 5a,b). No correlation was instead, observed between KCNMA1 expression and mir-211 (Figure 5c). 

According to the correlation analysis, as determined in the GSE14831 dataset, transfection of the HCT116 colon cancer cell line with miR-17-5p was associated with a marked downregulation of KCNMA1 expression (Figure 5d).

**Figure 6:**
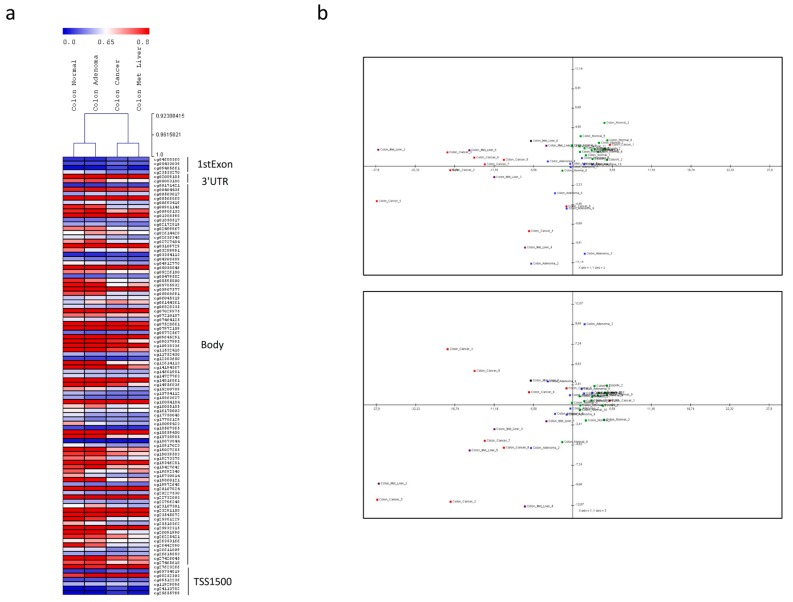
Methylation profiles of colorectal cancer samples as determined in the GSE53051 microarray dataset. (**a**) Methylation pattern of the *KCNMA1* gene; (**b**) Principal Component Analysis (PCA) of normal colon samples (*n* = 18, green samples), colon adenoma (*n* = 10, blue samples), colorectal adenocarcinoma (*n* = 9, red samples), and colorectal liver metastases (*n* = 6, violet samples). PCA graph was constructed using the first and second components (top panel) and the first and third components (bottom panel) for the KCNMA1-associated beta values.

The analysis of the methylation profile (Figure 6a) and the Principal Components Analysis (PCA) (Figure 6b, Table A1) show that the DNA methylation pattern of the *KCNMA1* gene allows to differentiate the normal colonic mucosa from adenoma and from adenocarcinoma, with and without liver metastases. However, as shown by PCA analysis, a high variance in the KCNMA1-associated methylation pattern can be observed among adenocarcinoma samples (Figure 6b). 

Interestingly, on the contrary to what can be observed in the promoter, numerous loci located in the body of the gene are significantly hypomethylated in the colon adenocarcinoma as compared to healthy tissue (Figure 6a). 

**Figure 7:**
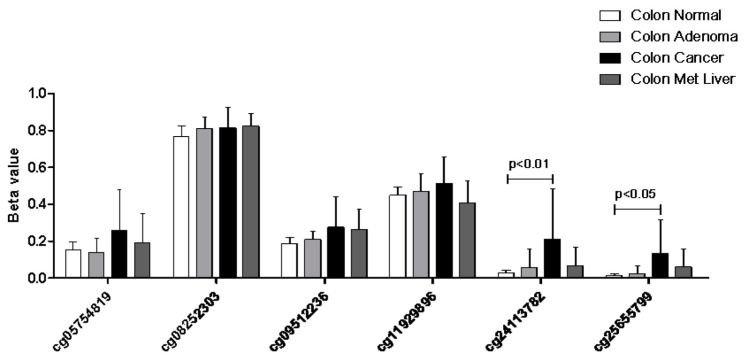
Methylation profiles of colorectal cancer samples as determined in the GSE53051 microarray dataset. Methylation patterns of the KCNMA1 promoter are expressed as Beta values.

When analyzing the promoter-associated methylation patterns, we found a significant hypermethylation of the cg24113782 and cg25655799 loci as compared to healthy tissue (Figure 7).

**Figure 8:**
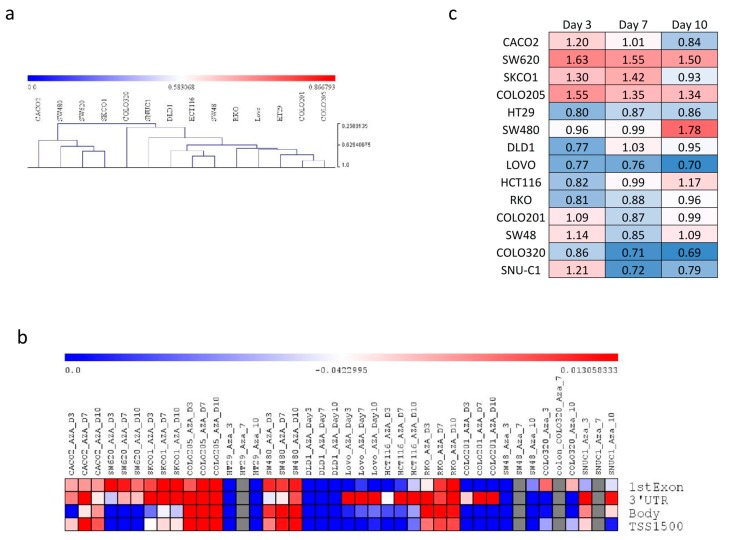
In vitro effect of the hypomethylating agent 5-azacitidine (AZA) on the expression of KCNMA1 in 14 human colon cancer cell lines. (**a**) Hierarchical clustering was performed on the KCNMA1-associated methylation levels, as obtained from the GSE57342 dataset; (**b**) heatmap showing the modulation of KCNMA1 gene methylation upon AZA treatment of 14 human colon cancer cell lines, as obtained from the GSE57342 dataset. The mean beta value is presented; (**c**) heatmap showing the modulation of KCNMA1 upon AZA treatment of 14 human colon cancer cell lines, as obtained from the GSE57341 dataset. The fold change variation respective to the untreated cells is presented.

In accordance with the high variability in the methylation patterns of the KCNMA1 gene observed in primary human samples, marked differences in KCNMA1 gene methylation were observed in established colon cancer cell lines (Figure 8a,b). Accordingly, treatment with the DNA-methyltransferase inhibitor 5-azacitidine (AZA) induced a variable degree of hypomethylation in the KCNMA1 gene promoter and body (Figure 8b). Therefore, treatment with AZA was associated with an increase in KCNMA1 expression in some colon cancer cell lines (i.e., SW620, SKCO1, COLO205, and SW480) (Figure 8c ), while a lack of effect, or even a downregulation of KCNMA1, was instead observed for other cells lines, such as LOVO and COLO320 (Figure 8c). 

## 3. Discussion

In the present study, we have studied the expression of KCNMA1 in preclinical and human CRC and found that *KCNMA1* is significantly downregulated in CRC, independently of the cancer stage. In addition, we propose that this downregulation is, at least partially, controlled by diverse epigenetic mechanisms, in particular DNA methylation and the miRNA, miR-17-5p. Our data are in line with observations from *Ma G* et al. [9] in gastric cancer, where it was found that the gene plays a tumor suppressor role. The lack of significant upregulation of KCNMA1 upon treatment of colon cancer cells with DAC can be explained in several ways. First of all, as shown in the heatmap presented in Figure 6, there is an opposite pattern of methylation in the promoter and body of the KCNMA1 gene. Indeed, while hypermethylation can be observed in the promoter, a general trend of hypomethylation can be observed in the gene body. Additionally, PCA analysis of the KCNMA1-associated methylation revealed a high variance among colon cancer samples, which is reflected by the high variability of methylation observed in the 14 colon cancer cell lines analyzed. Accordingly, a strong variability in KCNMA1 modulation is observed upon treatment of these cell lines with a DNA methyl-transferases inhibitor.

**Figure A1:**
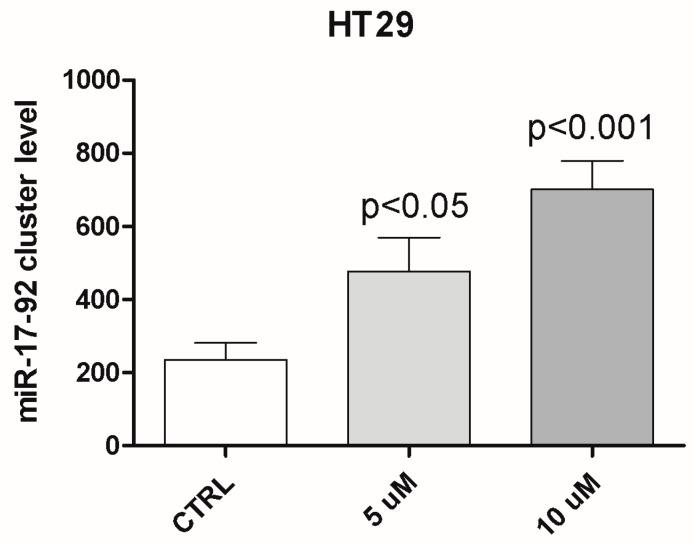
DAC treatment of HT29 cells determines an upregulation of the miR-17-92 cluster host gene.

Furthermore, additional mechanisms of gene expression regulation can take place; in particular, the role of miRNAs seems to be relevant, as shown in the paper. Interestingly, as shown in Appendix A Figure A1, DAC treatment of HT29 cells determines an upregulation of the miR-17-92 cluster host gene. This apparently contradictory result further supports the complex regulation of KCNMA1 expression in CRC.

Despite this, several other mechanisms underlying KCNMA1 expression are likely to be involved: for instance, histone modification (acetylation, methylation, sumoylation of the different core proteins); factors affecting RNA stability; transcription factors (e.g., at the gene enhancer GH10J077636 only, there seems to be putative transcription binding sites for 42 transcription factors: YY1, GLIS2, ZIC2, SP7, ZBTB8A, PRDM10, POLR2A, CTBP1, CTCF, ZEB2, PATZ1, ZNF524, BACH1, YBX1, REST, SCRT2, ZBTB20, EZH2, DPF2, ZNF561, ZBTB10, ZBTB26, IKZF1, NR2F1, OSR2, SMARCA5, ZBTB17, ZNF366, ZFHX2, RB1, ZNF335, ZNF777, GATA3, ZNF513, YY2, SP3, ZNF217, ZNF2, ZNF785 and EGR2, as shown in the GeneCards database, https://www.genecards.org/cgi-bin/carddisp.pl?gene=KCNMA1).

Our observation of the transcriptomic downregulation of KCNMA1 has also been confirmed by immunohistochemistry data from The Human Protein Atlas database and from immunohistochemistry analysis of the CRC samples available in the repository of the Department of Medical and Surgical Sciences and Advanced Technology “G.F. Ingrassia”, at the University of Catania. 

In epithelial cells of the gastrointestinal tract, KCNMA1 is implicated in a variety of cellular functions, e.g., electrolyte and substrate transport, cell volume regulation, cell migration, wound healing, proliferation, apoptosis, and carcinogenesis. Colonic electrolyte transport appears to be affected in the premalignant colon, which may contribute to the irregularities in defecation, often observed in patients with human colonic cancer. However, dysplastic transformation is associated with depolarization of the crypt epithelial cells, possibly indicating a change in K^+^ channel regulation or expression. However, the biological role of *KCNMA1* in the gastrointestinal tract is still to be deciphered. Ma and collaborators have previously shown that the overexpression of *KCNMA1* in the human gastric cancer cell lines, MGC803 and BGC823, was associated with a significant reduction in their invasion and migration properties and inhibited the tumor growth in a xenograft murine model [8]. They have also shown that KCNMA1 over-expression increased the numbers of both early apoptotic cells and late apoptotic cells in transfected MGC803 and BGC823 cells compared with control cells. In addition, they have shown an inverse correlation between KCNMA1 and the PTK2 gene, which is involved in FAK apoptosis pathways. Accordingly, KCNMA1 overexpression was not sufficient to inhibit the migration and invasion of gastric cancer cells after PTK2 knockdown [9]. 

On the other hand, in the metastatic breast cancer cell lines MCF7, MDA-MB-231, and MDA-MB-361, KCNMA1 promotes cell invasiveness and transendothelial migration, which could be attenuated by siRNA knockdown or inhibition with Iberiotoxin [5]. Further studies are therefore needed for a full understanding of the involvement of this gene in the development and progression of colon cancer. 

Another important finding of the study was the down-regulation of this gene observed in patients with colorectal adenocarcinoma with MSI as compared to healthy patients. The possibility of having a potential biomarker of MSI could represent an important step towards immunological targeting therapy, regardless of tumor histology and location. The MSI represents a prognostic and predictive factor in CRC: patients with a high MSI have a better prognosis with a good response to immunotherapy compared to those without MSI [15,16,17]. 

## 4. Materials and Methods

### 4.1. Animals

C57/BL6 male mice (Envigo, San Pietro al Natisone, UD, Italy) weighing between 20–22 g were housed within a limited access rodent facility and kept in groups of a maximum of five mice, in polycarbonate isolator cages with a filter top and external air supply and free access to food and water. Animal care was in compliance with local regulations on the protection of animals used for experimental and other scientific purposes (Directive 86/609/EEC, enforced by the Italian D.L. No. 116 of 27 January 1992).

### 4.2. Murine Models of UC and UC-Associated Cancer

The transcriptional levels of *KCNMA1* were initially determined in a preclinical model of UC, which was induced in the C57/BL6 mouse by the administration of 3% DSS in drinking water. The colon samples were collected at day 0, 2, 4, and 6 post-induction (*n* = 5–6 mice per group), for the analysis of *KCNMA1* expression. The well-established DSS/AOM model was used for UC-associated cancer. Briefly, a single dose of AOM (10 mg/kg) was administered intra-peritoneally to day 1, followed by three cycles of DSS in drinking water (cycle 1: 2%, days 8~14, cycle 2: 1.5%, day 29~33; cycle 3: 1.5%, day 50~54). Mucosa samples were collected at 2, 4, 8, and 20 weeks. 

### 4.3. RNA Isolation and Real-Time RT-PCR

Total RNA was extracted from samples using TRIzol reagent (Life Technologies, Monza, Italy). Two micrograms of RNA were retro-transcribed, and cDNA was used for the determination of *KCNMA1* by real-time RT-PCR using the FastStart SYBR Green Master (Roche, Monza, Italy). Primer sequences were in house-designed or obtained from the PrimerBank database (http://pga.mgh.harvard.edu/primerbank/).

### 4.4. Analysis of Transcription Profiles of KCNMA1 in Patients with CRC

The transcriptional levels of *KCNMA1* in CRC samples were evaluated in the two microarray datasets, GSE24514 and GSE32323, obtained from the Gene Expression Omnibus database (GEO; https://www.ncbi.nlm.nih.gov/gds). GSE24514 included expression data from 34 microsatellite-instable CRC samples and 15 normal colonic mucosal samples. MSI samples were of both sporadic and HNPCC (Hereditary Non-Polyposis Colorectal Cancer) origin and included ordinary and mucinous adenocarcinomas. Additionally, the samples consisted of right and left colons, as well as the rectum [15]. GSE24514 was generated using the Affymetrix Human Genome U133A Array and data were analyzed by MAS5.0 and normalized using the quantile normalization procedure. The GSE32323 included data from 17 pairs of cancer and non-cancerous tissues from CRC patients. Clinical and demographic data of the patients included in the study are available in the relative publication [16]. GSE32323 was generated using the Affymetrix Human Genome U133 Plus 2.0 Array and data were normalized by robust multichip analysis (RMA).

Data regarding the levels of *KCNMA1* in patients with CRC were obtained from the TGCA database, through the cBioPortal for Cancer Genomics (http://www.cbioportal.org/) website. RNA-seq data were processed and normalized using RSEM to generate TPM (transcripts per million). Overall, this study comprised RNA-seq data from 282 CRC samples. Forty samples consisted of mucinous adenocarcinoma of the colon and rectum. None of the patients received neoadjuvant therapy prior to resection. Identification of genetic aberrations in the *KCNMA1* genes has been performed on a cohort of 70 DNA-sequenced CRC patients, as available in the TGCA database.

### 4.5. Immunohistochemistry Analysis of KCNMA1 Expression in CRC Samples

Modulation of KCNMA1 expression in CRC was validated using data obtained from the Human Protein Atlas database (https://www.proteinatlas.org/pathology) and from immunohistochemical staining of a small number of CRC samples available in the repository of the Department of Medical and Surgical Sciences and Advanced Technology “G.F. Ingrassia”, University of Catania. Immunohistochemistry analyses were performed using the standard streptavidin–biotin labeling technique using the LSAB kit (Dako, Glostrup, Denmark) with appropriate positive and negative controls. Sections derived from paraffin embedded specimens, obtained from biopsies of three patients with normal colonic mucosa, three patients with tubule-villous adenomas, and three patients with invasive adenocarcinomas, were deparaffinized in xylene for 15 min, rehydrated, and treated with 3% H_2_O_2_ for 10 min to block endogenous peroxidase activity. Briefly, deparaffinized sections were incubated with anti-KCNMA1 antibodies (HPA054648, Sigma-Aldrich, Milan, Italy) (dilution: 1:400). Microwave pretreatment was crucial to enhancing the staining in all samples examined. Bound antibody was revealed by incubation with 3,3-diaminobenzidine (Sigma-Aldrich, St.Louis, MO, USA) in 0.01% H_2_O_2_ for 5 min at room temperature. Sections were then counterstained with hematoxylin, dehydrated, and mounted. Negative controls involving the omission of the primary antibody were included.

### 4.6. Analysis of Epigenetic Regulation of KCNMA1 Expression in CRC

The association between KCNMA1 expression and the levels of the miRNAs, mir-17-5p, mir-31, and mir-211 was determined for samples of patients with colorectal adenocarcinoma from the GSE35834, available in the GEO databank. The dataset included data from 31 primitive colorectal cancer from patients who underwent surgery at the University of Padova [18]. The Affymetrix Multispecies miRNA-1 Array and Affymetrix Human Exon 1.0 ST Array were used to generate the dataset. For both platforms, raw data were preprocessed using the RMA algorithm. For the miRNA-1 Array, only the human probes were considered for the normalization and summarization.

The DNA methylation patterns of the KCNMA1 gene were obtained from the GSE53051 microarray dataset. The dataset contains the methylation profiles of normal colon samples (*n* = 18), colon adenoma (*n* = 10), colorectal adenocarcinoma (*n* = 9), and CRC with liver metastases (*n* = 6) [19]. The platform used for the dataset was the Illumina Infinium 450k Human Methylation Beadchip.

In order to investigate the effect of the hypomethylating agent AZA on the expression and methylation levels of KCNMA1, the GSE57341 and GSE57342 datasets were interrogated [20]. These datasets include whole-genome expression and methylation data of 14 human colon cancer cells lines, treated with either 0 or 0.5 μM of AZA at different time points (day 3, 7, and 10). 

### 4.7. Statistical Analysis

Statistical differences between groups were evaluated by applying either the ANOVA or two-tailed Student’s T test. The correlation between KCNMA1 levels and OS was calculated using the non-parametric Spearman’s test. Values of *p* < 0.05 were considered statistically significant.

## 5. Conclusions

The characterization of the expression levels of KCNMA1 and the mechanisms underlying its modulation could help in defining its role in the CRC. Overall, our data suggest the possible use of KCNMA1 as a biomarker in the early stages of CRC and as a possible therapeutic target. 

## Figures and Tables

**Table 1:** Mutations of KCNMA1 in colorectal cancer patients.

Sample ID	Cancer Type	Protein Change	Mutation Type	Copy #
TCGA-AA-3831-01	Colon Adenocarcinoma	S60del	IF del	Diploid
TCGA-AA-3524-01	Colon Adenocarcinoma	T905M	Missense	Diploid
TCGA-AA-A01V-01	Colon Adenocarcinoma	R909W	Missense	Diploid
TCGA-AA-A00E-01	Colon Adenocarcinoma	S580P	Missense	Diploid
TCGA-AA-A00L-01	Colon Adenocarcinoma	A381T	Missense	Diploid
TCGA-AA-3984-01	Colon Adenocarcinoma	S1190P	Missense	Diploid
TCGA-AA-A00N-01	Mucinous Adenocarcinoma of the...	K1153T	Missense	Diploid
TCGA-AA-A00N-01	Mucinous Adenocarcinoma of the...	N801S	Missense	Diploid
TCGA-AA-A010-01	Colon Adenocarcinoma	L1236F	Missense	Diploid
TCGA-AA-A010-01	Colon Adenocarcinoma	E824D	Missense	Diploid

## Appendix A

**Table A1:** PCA coordinates for the samples in GSE53051.

Sample	X	Y	Z
Colon_Normal_1	1.735467	1.358078	−1.74759
Colon_Normal_2	4.342013	5.111035	−1.65102
Colon_Normal_3	−0.25661	2.49674	0.359764
Colon_Normal_4	1.501905	2.161722	0.570626
Colon_Normal_5	0.961287	3.563164	0.071522
Colon_Normal_6	3.428178	1.750228	1.24613
Colon_Normal_7	3.580552	2.792617	1.531011
Colon_Normal_8	−1.62841	−0.42259	−4.3392
Colon_Normal_9	4.737718	3.108791	1.336153
Colon_Normal_10	0.88916	1.758846	−0.51744
Colon_Normal_11	0.849407	2.382122	1.14948
Colon_Normal_12	2.52812	1.950024	0.647312
ColonN_2	2.949582	2.091265	2.387948
ColonN_2	4.124992	1.94098	0.049728
ColonN_2	4.724679	0.837779	2.424594
ColonN_2	4.442956	2.109996	1.577544
ColonN_2	2.655884	0.930409	1.088807
Colon_Adenoma_1	1.481603	0.340708	1.481484
Colon_Adenoma_2	−6.04664	−11.1386	−5.05503
Colon_Adenoma_3	1.561727	−9.99486	9.808415
Colon_Adenoma_4	−3.68738	0.668282	2.941357
Colon_Adenoma_5	−0.52092	0.373219	1.291463
Colon_Adenoma_6	−2.653	−3.03014	−0.95898
Colon_Adenoma_7	−0.44732	−0.00646	−0.31317
Colon_Adenoma_8	1.141704	1.037116	2.029349
Colon_Adenoma_9	−1.0016	−4.7615	−1.04647
Colon_Adenoma_10	2.62769	0.301795	1.581635
Colon_Cancer_1	5.112652	2.56774	0.636602
Colon_Cancer_2	−17.4902	−0.3567	−11.5103
Colon_Cancer_3	−17.9117	1.695159	6.819301
Colon_Cancer_4	−6.336	−7.3712	1.640066
Colon_Cancer_5	−27.9047	−3.92621	−11.265
Colon_Cancer_6	−1.03183	−4.53172	2.102261
Colon_Cancer_7	−12.6164	0.340978	−4.18775
Colon_Cancer_8	−9.90718	0.857501	−5.01775
Colon_Cancer_9	−14.0838	1.098967	4.202911
Colon_Met_Liver_1	−3.8611	2.457655	−1.83603
Colon_Met_Liver_2	−27.6862	2.011681	−9.32142
Colon_Met_Liver_3	−11.2724	−1.14682	−2.78756
Colon_Met_Liver_4	−6.92045	−9.26846	−12.0695
Colon_Met_Liver_5	−14.7814	1.954306	−5.35754
Colon_Met_Liver_6	−6.07612	3.006146	3.031533
